# Link between Domoic Acid Production and Cell Physiology after Exchange of Bacterial Communities between Toxic *Pseudo-nitzschia multiseries* and Non-Toxic *Pseudo-nitzschia delicatissima*

**DOI:** 10.3390/md12063587

**Published:** 2014-06-11

**Authors:** Aurélie Lelong, Hélène Hégaret, Philippe Soudant

**Affiliations:** Marine Environmental Sciences Laboratory (Laboratoire des sciences de l’environnement marin, LEMAR), UMR6539, European Institute for Marine Studies (Institut Universitaire Européen de la Mer, IUEM), Rue Dumont d’Urville, Plouzané 29280, France; E-Mails: aurelai86@hotmail.com (A.L.); philippe.soudant@univ-brest.fr (P.S.)

**Keywords:** bacteria, domoic acid, physiology, *Pseudo-nitzschia*

## Abstract

Bacteria are known to influence domoic acid (DA) production by *Pseudo-nitzschia* spp., but the link between DA production and physiology of diatoms requires more investigation. We compared a toxic *P. multiseries* to a non-toxic *P. delicatissima*, investigating links between DA production, physiological parameters, and co-occurring bacteria. Bacterial communities in cultures of both species were reduced by antibiotic treatment, and each of the diatoms was inoculated with the bacterial community of the other species. The physiology of *P. delicatissima* was minimally affected by the absence of bacteria or the presence of alien bacteria, and no DA was detected. *P. multiseries* grew faster without bacteria, did not produce a significant amount of DA, and exhibited physiological characteristics of healthy cells. When grown with alien bacteria, *P. multiseries* did not grow and produced more DA; the physiology of these cells was affected, with decreases in chlorophyll content and photosynthetic efficiency, an increase in esterase activity, and almost 50% mortality of the cells. The alien bacterial community had morphological and cellular characteristics very different from the original bacteria, and the number of free-living bacteria per algal cell was much higher, suggesting the involvement of bacteria in DA production.

## 1. Introduction

In 1987, three people died after ingesting blue mussels contaminated with domoic acid (DA), a neurotoxin causing amnesic shellfish poisoning [1]. DA is produced by species of the diatom genus *Pseudo-nitzschia*, but only some species, and even strains of some species, are toxic while others are not (reviewed in [2]). Since 1987, many studies have been published trying to understand when and why this toxin is produced (reviewed in [2]). Numerous factors can modulate DA production by *Pseudo-nitzschia* spp., including: macronutrient concentration [3], nitrogen source [4], micronutrient availability [5], growth phase [3,6], bacterial community [7], or even the age of a cultured isolate [8]. Nevertheless, no study to date has succeeded in explaining the role of DA in *Pseudo-nitzschia* physiology and ecology and how all the external factors can modulate DA production. The lack of comprehensive understanding stems in part from the fact that parameters measured often are limited to DA production, growth, and sometimes chlorophyll content or photosynthetic rate [9]. Very few physiological measurements have been performed to better understand the physiological state of the cell relative to DA production.

Thus, to manipulate *Pseudo-nitzschia* physiology, we modified the bacterial population associated with two cultured *Pseudo-nitzschia* species. In diatoms, growth, extracellular polymeric substances, secreted carbohydrates and proteins, or aggregation capacity, can be influenced by co-occurring bacteria [10,11,12]. Moreover, it has been shown that axenic cultures of *P. multiseries* produced no detectable or exceedingly low levels of DA and that the reintroduction of bacteria enhanced this production by 2- to 95-fold [7,13,14,15]. In one such study, all the bacterial communities tested, even from *Chaetoceros* sp. cultures, enhanced the production of DA by toxic *Pseudo-nitzschia* species [7]. Several hypotheses were provided to explain the role of bacteria in DA production, but these were not supported by cause-effect relationships.

To link DA production and physiological state, we exchanged the free-living bacterial community from a toxic strain of *P. multiseries* with that of a non-toxic strain of *Pseudo-nitzschia delicatissima*. Axenic and xenic cultures were compared, in terms of DA production, physiological state, and metabolic processes. Indeed, the acquisition of energy by cells starts with photosynthesis, which was assessed by measuring the quantum yield (efficiency of photosynthesis at the photosystem II level) and the chlorophyll content (estimated by cell autofluorescence). Esterase activity was estimated by measuring of FDA hydrolysis [16,17,18], a proxy of primary metabolism. Measurement of DA production was used as an indicator of the secondary metabolism in *P. multiseries*. Storage of extra energy in the form of lipid was measured using the BODIPY 493/503 probe [17,18,19]. Thus, allocation of energy to primary metabolism and cell division, secondary metabolism, or energy storage could be monitored during the progression of the diatom culture. Cell death was also monitored, using the SYTOX Green probe [17,18,19]. Bacteria were counted and their survival over time was also monitored [19]. These measurements allowed us to investigate *Pseudo-nizschia* cell responses to different bacterial communities and to determine how DA production and physiology were modified depending on the added bacterial community.

## 2. Results

### 2.1. Algal Growth and Death

**Figure 1:**
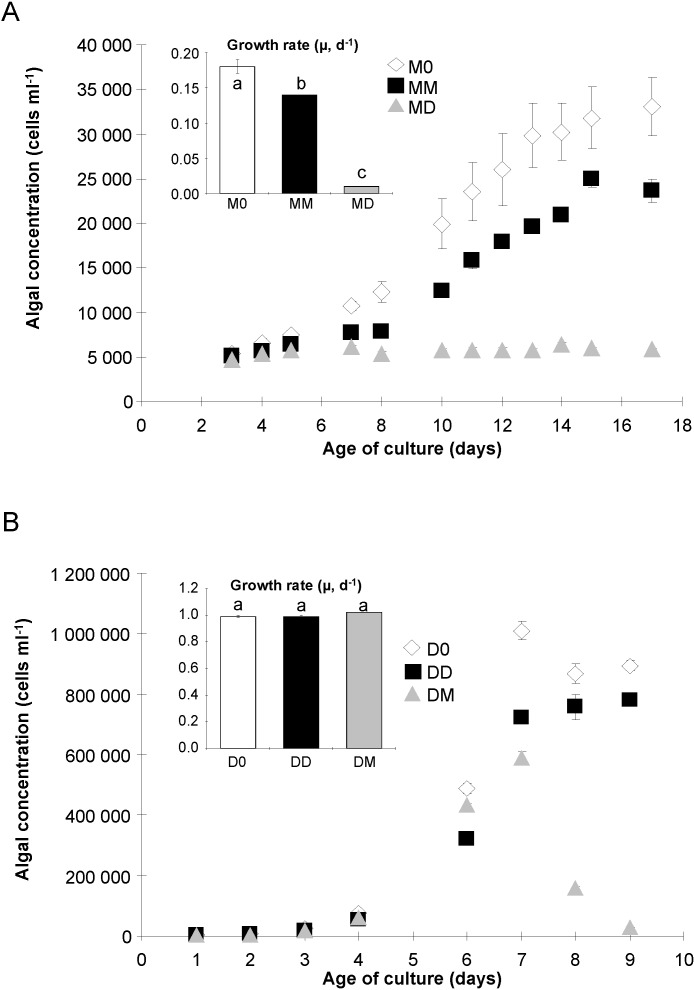
Population density (cells mL^−1^) of *P. multiseries* (**A**) and *P. delicatissima* (**B**) without added bacteria (M0 and D0, open diamonds), with the bacterial community of *P. multiseries* (MM and DM, filled squares) or with the bacterial community of *P. delicatissima* (MD and DD, grey triangles). Inserts show the specific growth rate µ (day^−1^) of each species. Letters indicate significantly different values (*p* < 0.05). Mean ± SE, *n* = 3.

Cells of *P. multiseries* exhibited differences in growth rate depending on the treatment (Figure 1A). Cultures with no added bacteria (M0) grew faster than those with *P. multiseries* bacteria added back (MM, *p* < 0.05), but cultures with *P. delicatissima* bacteria (MD) did not grow (Figure 1A). Cultures also reached significantly (*p* < 0.05) different cell densities, with the fastest growing culture (M0) reaching the highest population (Figure 1A). On the contrary, cultures of *P. delicatissima* did not exhibit any significant differences in growth rate in exponential phase (*p* > 0.05) attributable to experimental treatment (Figure 1B). Moreover, the *P. delicatissima* cultures reached stationary phase earlier (on day 7) than did the *P. multiseries* cultures; monitoring was thus stopped after day 9.

D0 cultures reached a higher mean cell concentration and maintained it for longer than did DD cultures (*p* < 0.05, Figure 1B). DM cultures reached the lowest maximal cell concentration and did not exhibit any stationary phase, with cell concentration decreasing immediately after the exponential phase ended (Figure 1B).

**Figure 2:**
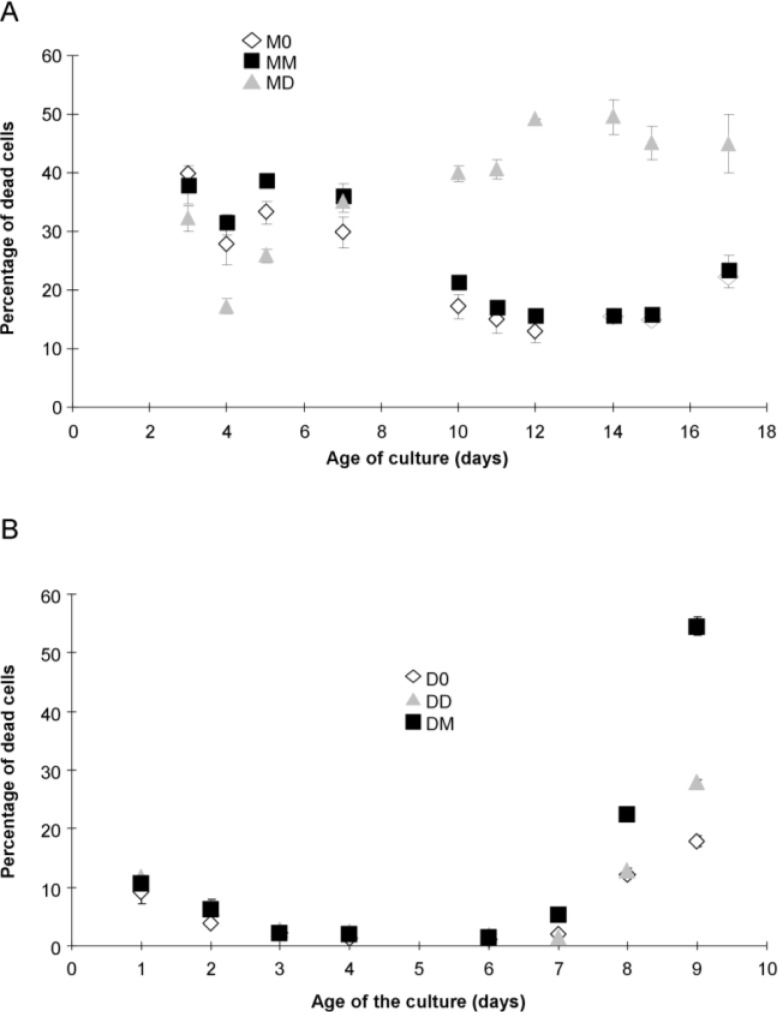
Percentage of dead *P. multiseries* (**A**) and *P. delicatissima* (**B**) cells, as determined by SYTOX Green staining: without added bacteria (M0, open diamonds), with the bacterial community of *P. multiseries* (MD, filled squares) or with the bacterial community of *P. delicatissima* (MM, grey triangles). Mean ± SE, *n* = 3.

The percentage of *P. multiseries* cells stained by SYTOX Green (permeable cells considered to be dead) was high and almost the same for the three treatments until day 7 (*p* > 0.05, Figure 2A). After day 7, cultures MM and M0 recovered, having a lower percentage of dead cells; whereas, almost 50% of the diatom cells in the MD cultures were dead (Figure 2A).

The percentage of dead algal cells decreased in all *P. delicatissima* cultures between days 1–6 and was not significantly (*p* > 0.05) different between treatments (Figure 2B). After day 7, DM cultures began to die faster than did the DD cultures (at the end of stationary phase); whereas, D0 exhibited the lowest percentage of dead cells (Figure 2B).

The percentage of active *P. multiseries* cells increased between days 3–4 for all cultures and remained identical until day 7 (Table 1). From day 7, the percentage of active MD cells decreased and was lower than for M0 and MM cells (Table 1).

The percentage of active *P. delicatissima* cells was the same regardless of the treatment until day 9 when DM cultures had fewer active cells than DD cultures; D0 cultures had the highest percentage of active cells (Table 1).

**Table 1:** Physiological parameters of *P. multiseries* and *P. delicatissima* cells grown without added bacteria (M0 and D0, respectively), with its own bacteria (MM and DD, respectively) or with bacteria from the other *Pseudo-nitzschia* species (MD and DM, respectively). Percentage of active cells and esterase activity were measured after FDA staining and lipid content after BODIPY staining, the latter two expressed in arbitrary units.

**Physiological Parameter**	**Age of Culture (Days)**	**Mean ± SE**	**Mean ± SE**	**Mean ± SE**
		***P. multiseries***		
		**M0**	**MM**	**MD**
**Percentage of Active Cells**	3	76.9 ± 2.0	73.9 ± 1.2	81.1 ± 0.8
	4	97.6 ± 1.4	95.5 ± 1.1	93.6 ± 1.5
	5	97.2 ± 0.8	98.3 ± 0.4	95.8 ± 1.0
	7	97.3 ± 0.2	96.6 ± 0.5	95.2 ± 0.4
	8	96.7 ± 0.4	95.7 ± 0.2	93.2 ± 0.8
	10	97.3 ± 0.4	97.7 ± 0.1	95.0 ± 0.7
	11	97.6 ± 0.4	98.0 ± 0.2	92.8 ± 1.5
	12	97.5 ± 0.2	97.0 ± 0.5	92.1 ± 1.2
	14	97.3 ± 0.3	98.1 ± 0.2	90.7 ± 1.5
	15	96.5 ± 0.2	98.1 ± 0.7	92.8 ± 0.7
	17	94.4 ± 0.4	96.2 ± 1.2	85.9 ± 2.9
		**M0**	**MM**	**MD**
**Esterase Activity**	3	130.0 ± 7.8	149.3 ± 13.2	138.7 ± 20.8
	4	157.3 ± 37.3	132.7 ± 11.4	167.2 ± 27.7
	5	76.8 ± 45.5	114.2 ± 8.5	187.1 ± 15.3
	7	122.5 ± 8.3	139.5 ± 6.4	198.5 ± 21.5
	8	116.3 ± 3.6	129.4 ± 7.9	159.9 ± 7.4
	10	121.6 ± 1.6	148.9 ± 9.0	145.2 ± 15.1
	11	112.4 ± 5.1	137.3 ± 8.7	148.5 ± 9.0
	12	111.1 ± 1.5	128.7 ± 9.7	141.9 ± 9.9
	14	126.6 ± 9.2	161.6 ± 7.6	128.5 ± 15.0
	15	106.2 ± 7.4	140.7 ± 11.5	114.1 ± 7.7
	17	131.9 ± 15.6	174.0 ± 13.8	111.9 ± 15.6
		**M0**	**MM**	**MD**
**Lipid Content**	3	1466.4 ± 65.6	1443.7 ± 42.7	1303.8 ± 48.4
	4	1516.1 ± 123.7	1572.0 ± 212.6	1493.2 ± 293.8
	5	1530.4 ± 83.2	1336.8 ± 171.1	1353.7 ± 23.5
	7	1099.5 ± 123.6	1099.4 ± 255.4	1147.7 ± 38.0
	8	1121.6 ± 63.7	1193.9 ± 67.1	1050.7 ± 17.1
	10	1013.9 ± 41.5	1092.6 ± 30.8	961.0 ± 19.2
	11	907.0 ± 39.5	1019.8 ± 11.4	878.9 ± 45.9
	12	826.9 ± 33.0	999.3 ± 11.6	912.5 ± 141.7
	14	913.2 ± 81.8	1240.1 ± 20.7	982.0 ± 70.3
	15	822.7 ± 16.3	984.0 ± 37.2	794.7 ± 30.1
	17	785.7 ± 28.7	989.5 ± 3.7	730.0 ± 43.5
**Physiological Parameter**	**Age of Culture (Days)**	**Mean ± SE**	**Mean ± SE**	**Mean ± SE**
		***P. delicatissima***		
		**D0**	**DD**	**DM**
**Percentage of Active Cells**	1	96.0 ± 0.5	95.4 ± 1.3	95.3 ± 1.5
	2	98.9 ± 0.3	98.4 ± 0.4	98.7 ± 0.3
	3	99.6 ± 0.2	99.2 ± 0.2	99.1 ± 0.2
	4	99.5 ± 0.1	99.0 ± 0.4	99.2 ± 0.0
	6	99.4 ± 0.2	98.6 ± 0.1	98.9 ± 0.1
	7	99.3 ± 0.1	99.0 ± 0.1	99.0 ± 0.0
	8	98.9 ± 0.1	98.4 ± 0.0	98.5 ± 0.1
	9	97.5 ± 0.1	94.9 ± 0.0	86.7 ± 0.5
		**D0**	**DD**	**DM**
**Esterase Activity**	1	33.6 ± 3.0	37.9 ± 2.4	35.1 ± 2.3
	2	46.5 ± 0.4	51.1 ± 4.6	52.0 ± 3.4
	3	33.9 ± 0.2	40.9 ± 1.0	38.8 ± 2.0
	4	34.9 ± 1.8	37.8 ± 4.5	40.5 ± 0.8
	6	40.1 ± 4.4	33.1 ± 0.8	45.6 ± 5.4
	7	47.4 ± 0.2	38.2 ± 1.7	60.2 ± 2.0
	8	33.4 ± 1.6	38.9 ± 1.4	73.0 ± 15.7
	9	34.2 ± 1.6	45.4 ± 1.0	59.5 ± 9.5
		**D0**	**DD**	**DM**
**Lipid Content**	1	108.5 ± 20.9	102.7 ± 5.3	105.6 ± 4.2
	2	124.9 ± 13.6	138.4 ± 27.3	128.8 ± 23.9
	3	116.3 ± 3.7	151.9 ± 7.4	135.8 ± 4.4
	4	124.9 ± 4.3	161.8 ± 4.6	152.0 ± 15.3
	6	150.3 ± 11.6	227.6 ± 10.7	184.2 ± 9.6
	7	95.5 ± 13.2	158.6 ± 8.3	148.8 ± 5.5
	8	118.9 ± 17.3	143.7 ± 1.6	171.8 ± 3.5
	9	207.7 ± 10.6	199.8 ± 5.6	242.9 ± 4.7

### 2.2. Bacterial Growth

**Figure 3:**
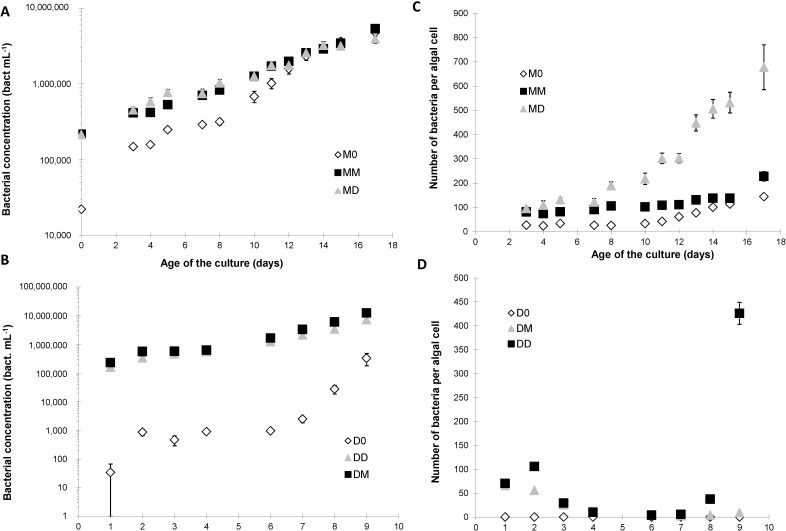
Concentration (bacteria mL^−1^, **A**,**B**) and number of free-living bacteria per algal cell (**C**,**D**) of free-living bacteria associated with *P. multiseries* (**A**,**C**) and *P. delicatissima* (**B**,**D**) without added bacteria (M0 and D0, open diamonds), with the bacterial community of *P. multiseries* (MM and DM, filled squares) or with the bacterial community of *P. delicatissima* (MD and DD, grey triangles). Mean ± SE, *n* = 3.

**Figure 4:**
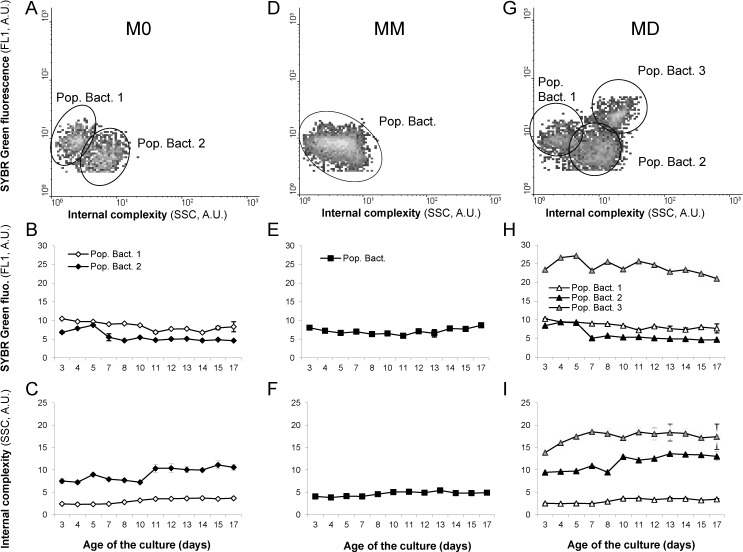
Flow cytometric determinations (in arbitrary units; A.U.) of internal complexity (as measured by Side Scatter “SSC”) and DNA content (as estimated by SYBR Green I fluorescence) of bacteria from M0 (**A**–**C**; containing 2 populations), from MM (**D**–**F**; containing 1 population) and from MD (**G**–**I**; containing three populations) and their variations/dynamics during culture (**B**,**C**,**E**,**F**,**H**,**I**). Mean ± SE, *n* = 3.

Despite the AB treatment, M0 cultures still contained bacteria, although initially 4-fold fewer than in the MM and MD cultures, which had the same bacterial concentration (Figure 3A). Bacteria in the M0 cultures grew faster than those in the MD and MM cultures and reached concentrations equivalent to those in the MD and MM bacterial cultures on day 12 (Figure 3A). The morphological characteristics of bacteria from the three cultures were, however, quite different from each other during the entire growth phase (Figure 4). In the M0 culture, two populations of bacteria could be defined (Figure 4A–C), based on some morphological (cell internal complexity) and cellular (DNA content) parameters; whereas, only one population of bacteria could be distinguished in the MM cultures (Figure 4D–F). In the MD cultures, three different populations of bacteria could be discriminated, with the third population (“Pop. Bact. 3”) having very different morphological (internal complexity) and cellular (DNA content) (Figure 4G–I) than the other bacterial populations.

The D0 culture of *P. delicatissima* initially was “quasi axenic,” with almost no bacteria until day 6, after which bacteria started to grow (Figure 3B). The DD culture had significantly (*p* < 0.05) fewer bacteria ((7.3 ± 0.1) × 10^6^ bacteria mL^−1^) than the DM culture ((12.3 ± 0.2) × 10^6^ bacteria mL^−1^) on day 9 (Figure 3B).

The number of free-living bacteria per algal cell remained within the same range (<200) during the entire culture cycle in the MM and M0 cultures (Figure 3C). The number of free-living bacteria per algal cell in the MD culture (Figure 3C), however, started to increase drastically on day 7, reaching ~700 free-living bacterial per *P. multiseries* cell at the end of the stationary phase on day 17.

In *P. delicatissima* DM and DD cultures (Figure 3D), the number of free-living bacteria per algal cell also remained similar (<150) during the entire culture cycle, except in the DD culture, on day 9 when cells began dying (Figure 2B). This remained very low during the entire culture cycle in the D0 cultures (Figure 3D).

### 2.3. Domoic Acid

**Figure 5:**
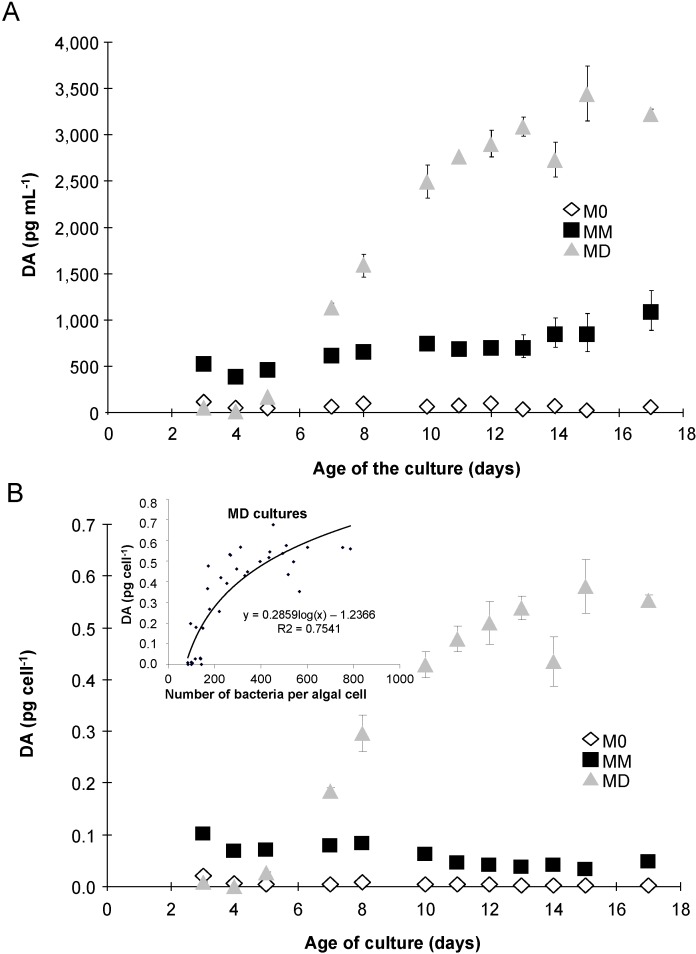
Total domoic acid (DA), as pg mL^−1^ (**A**) or pg cell^−1^ (**B**), in *P. multiseries* cultures grown without added bacteria (M0, open diamonds), with the bacterial community of *P. multiseries* (MM, filled squares) or with the bacterial community of *P. delicatissima* (MD, grey triangles). Mean ± SE, *n* = 3. Insert shows the significant relationship between “whole culture” DA per cell and the number of free-living bacteria per algal cell in the MD cultures (*n* = 35, *p* < 0.0001).

**Figure 6:**
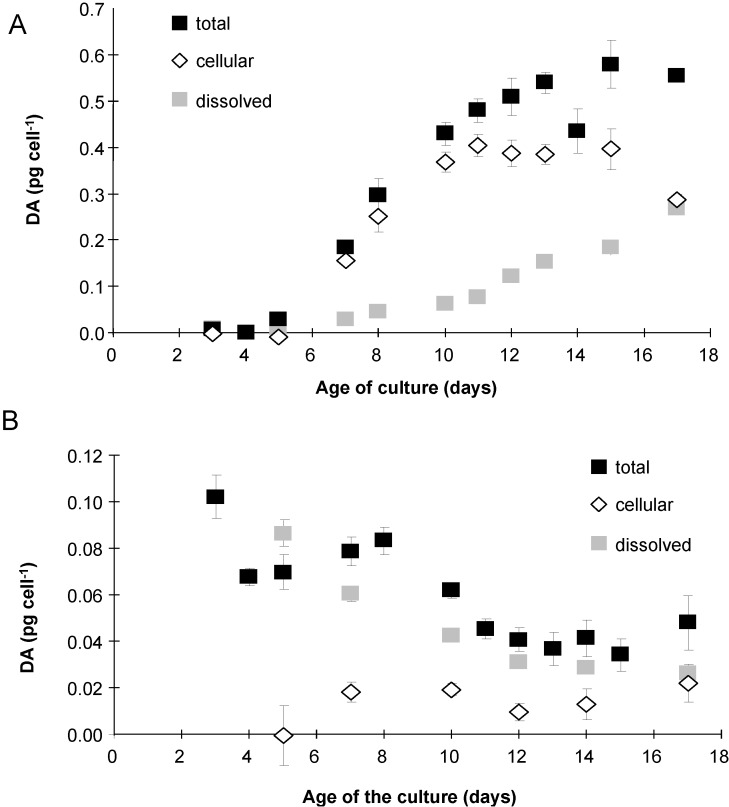
Total (filled squares), cellular (open diamonds) and dissolved (grey squares) domoic acid (DA) in *P. multiseries* cultures grown with (**A**) *P. delicatissima* bacteria (MD) or (**B**) *P. multiseries* bacteria (MM). Mean ± SE, *n* = 3.

M0 cultures exhibited very low levels of DA per mL of culture (<130 pg DA mL^−1^), indicating little production of DA (Figure 5A). On the other hand, MD and MM cultures produced DA, with the concentration of DA increasing with the age of the culture (Figure 5A,B). M0 cultures exhibited <0.01 pg DA cell^−1^ in the whole culture (Figure 5B), regardless of the growth phase; thus, dissolved and intracellular DA were not measured. MD cultures had significantly more DA per cell than did the MM cultures (*p* < 0.05). MD and MM cultures also exhibited a different pattern of production, expressed as either DA per cell or per mL, which increased drastically after day 7 in the MD cultures (Figure 5A,B). Cells in the MD cultures did not grow (Figure 1A), but DA per cell increased up to 17-fold more than that found in the MM cultures (Figure 5B), *i.e.*, from 0.18 to 0.58 pg cell^−1^ in the MD cultures from day 7 to day 15; whereas, the MM cultures decreased from 0.08 to 0.03 pg cell^−1^. In MD cultures, a significant relationship was observed between the total DA per cell and the number of free-living bacteria per algal cell (insert, Figure 5B, with logarithmic regression: Y = 0.2859 log(X) − 1.2366, *R*^2^ = 0.7541, *p* < 0.0001). In MD cultures, almost all the DA was stored within the cells (80%) until day 11, after which the cells started to release DA into the medium, reaching 50% of the total DA (Figure 6A). In contrast, almost all the DA contained in the MM cultures was found in the dissolved fraction (Figure 6B). Cultures of *P. delicatissima* did not exhibit any measurable dissolved or intracellular DA, regardless of the treatment and growth phase [20].

### 2.4. Photosynthetic Parameters

**Figure 7:**
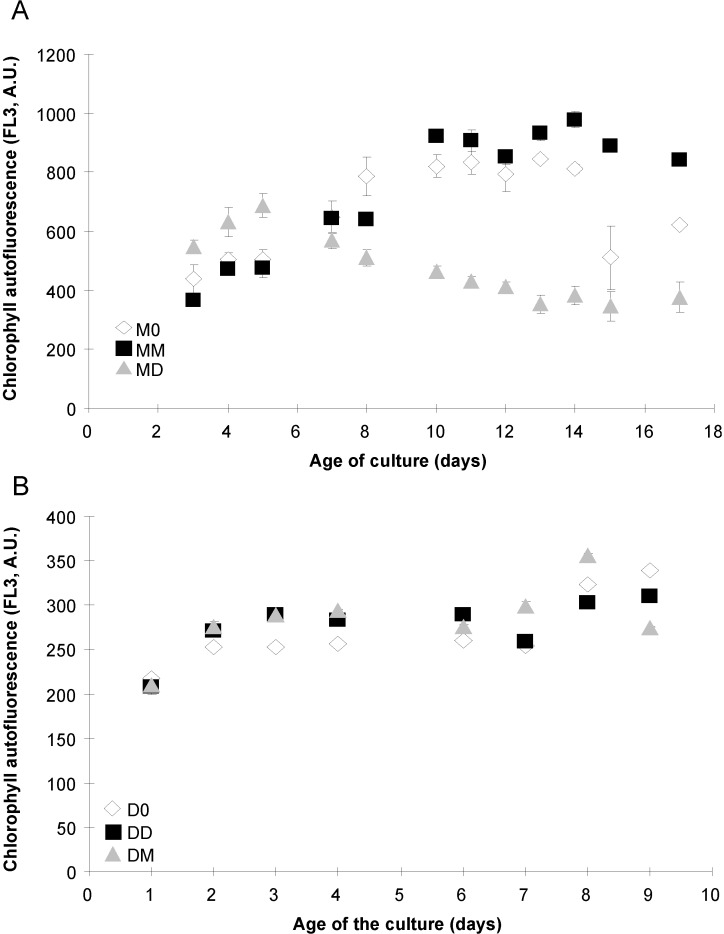
Cell autofluorescence (FL3, in arbitrary units, A.U.), related to chlorophyll content of *P. multiseries* (**A**) and *P. delicatissima* (**B**) grown without added bacteria (M0 and D0, open diamonds), with the bacterial community of *P. multiseries* (MM and DM, filled squares) or with the bacterial community of *P. delicatissima* (MD and DD, grey triangles). Mean ± SE, *n* = 3.

**Figure 8:**
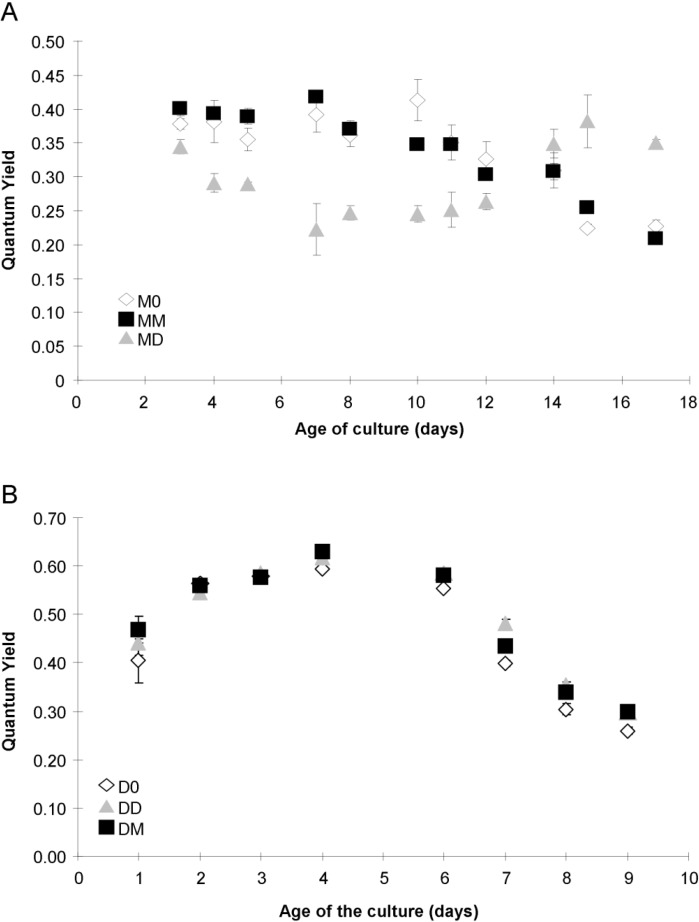
Quantum Yield (QY) at the PSII level for *P. multiseries* (**A**) and *P. delicatissima* (**B**) over time, grown without added bacteria (D0 and M0, open diamonds), with the bacterial community of *P. multiseries* (DM and MM, filled squares) or with the bacterial community of *P. delicatissima* (MD and DD, grey triangles). Mean ± SE, *n* = 3.

Cell autofluorescence of MD cultures decreased after day 5 (Figure 7A) when cells stopped growing (Figure 1A). FL3 values of M0 and MM cultures were similar and increased during the exponential phase to reach significantly higher values than for MD cultures after day 8 (Figure 7A). Cultures M0 and MM had the same values of QY during the experiment (*p* > 0.05), with a decrease after day 7 until the end of the experiment (Figure 8A). Cultures of MD had a lower QY than did the M0 and MM cultures until day 12, after which they exhibited an increase in QY values until day 15, with higher values than the MM and M0 cultures (Figure 8A).

All the *P. delicatissima* cultures exhibited a similar increase in cell autofluorescence during the exponential growth phase, except for days 2–4, when D0 had a significantly lower autofluorescence than the DD and DM cultures (Figure 7B). Cell autofluorescence remained stable thereafter. The QY of *P. delicatissima* (D0, DD and DM) increased during the early exponential phase, and was similar regardless of the treatment. During the decrease in QY, D0 cultures had a slightly (but significantly, *p* < 0.05) lower QY than the DD and DM cultures, with all following the same pattern (Figure 8B).

### 2.5. Esterase Activity and Lipid Content

Esterase activity (hydrolysis of FDA) was significantly lower (*p* < 0.05) for M0 cultures than for MM cultures, although the same temporal pattern of activity was exhibited by both. On the other hand, MD cultures had a substantially different pattern of activity, with an increased esterase activity during the first days of culture (Table 1).

The three *P. delicatissima* cultures (D0, DD and DM) had the same peak of esterase activity on day 2. During stationary phase, when D0 cultures were dying, they had a significantly higher (*p* < 0.05) FDA fluorescence than did the DD and DM cultures, and thus higher esterase activity (Table 1).

BODIPY fluorescence of *P. multiseries* cells (a proxy for lipid content) followed the same pattern regardless of the treatment, with a decrease in lipid content during stationary phase (even for the MD culture that did not grow). The lipid content of the MM cultures was higher than that of the M0 and MD cultures; whereas, that of the M0 and MD cultures was not significantly different (Table 1).

The lipid content of *P. delicatissima* exhibited a totally different pattern; lipid content increased during the exponential phase and decreased at the beginning of the stationary phase, finally increasing during stationary phase, regardless of the treatment (Table 1). The D0 cultures had a significantly (*p* < 0.05) lower mean lipid content than the DM and DD cultures (Table 1).

## 3. Discussion

Results of this study indicate that changes in co-occurring bacteria can be detrimental to *Pseudo-nitzschia* cultures, and that increased DA production is associated with several physiological impairments. Both *Pseudo-nitzschia* species had been growing for at least two years in culture before these experiments were performed and were thus adapted to “their” bacterial communities. Following an immediate, but transient effect of AB on *P. delicatissima* cell physiology a good growth rate of 1.0 day^−1^, which represents ~2 generations per day, was sustained. A high percentage of dead cells was observed on day 1, perhaps attributable to cells permeabilized by AB. However, only a portion of the SYTOX Green-stained cells were really dead at this stage, as indicated by the high percentage of active cells. Approximately 20% of the cells were dead in untreated cultures of *P. multiseries* [19], which may be attributable to the age of the isolate. Nonetheless, both species experienced the same AB treatment, thus the cultures with and without added bacteria can be compared.

After treating *P. multiseries* cultures with AB, some bacteria survived and were able to grow during the experiment. Thus, M0 cultures were not axenic, and in MD cultures, bacterial communities associated with both *P. multiseries* and *P. delicatissima* co-existed. Nevertheless, the aim to modulate DA production by modifying the bacterial community and its concentration was achieved. Indeed, the addition of the bacterial community associated with *P. delicatissima* resulted in a 6- to 17-fold higher DA concentration per *P. multiseries* cell compared to *P. multiseries* cells grown with their “own” bacterial community. Moreover, in M0 cultures, DA production was decreased by almost 10-fold (to <0.02 pg cell^−1^) relative to the control culture MM. Decreased DA production in axenic cultures has been shown in previous studies [7,21,22]. Nevertheless, our cultures were not axenic, and it appeared that the removal of all the bacteria was not necessary to limit DA production. Moreover, in our study, we considered only free-living bacteria without taking into account attached bacteria [23]. In this study, some of these attached bacteria may have been killed with the AB treatment. When considering free-living bacteria, not only the bacterial concentration but also the bacterial composition may play a role in DA regulation. Sison-Mangus *et al.* [24] demonstrated that different bacterial communities were associated with higher production of DA. They also suggested that elevated DA production is a mere stress response to foreign bacteria, similar to any another environmental stress [24]. In contrast, our study suggests that alien bacteria seem to trigger the production of DA, as observed by the significant relationship between DA production and the number of free-living bacteria per algal cell. Although it is not possible to establish whether or not there is a direct or indirect relationship, the appearance of a new bacterial population with different morphological and cellular characteristics in MD cultures (“Pop. Bact.3”, Figure 4G–I) is speculated to be responsible for the induction of DA production. Several authors also hypothesized that only alien bacteria seem to provoke high production of DA, potentially viewed as a defense mechanism of toxic *Pseudo-nitzschia* cells against alien bacteria [7,23]. This could be explained if only some, specific bacteria induce DA production, and that these may have been killed by the AB treatment. Indeed, Osada and Stewart [22] hypothesized that only bacteria producing gluconolactone/gluconic acid are able to induce DA production, and that this could be attributable to the very powerful sequestering ability of gluconolactone/gluconic acid, which could act as a chelator of metals. Kaczmarska *et al.* [23] proposed that a specific bacterial community composition, or its concentration, may be important in explaining the variable DA levels associated with some *P. multiseries* cultures. They hypothesized that some bacteria (especially alien strains) were antagonistic to the algae and induced an increase in DA production as a specific response to the presence of bacteria. Kaczmarska *et al.* [23] also discussed the possible importance of attached bacteria, which may or may not have been killed by the AB treatment in this study. Nevertheless, our strain of *P. delicatissima* was confirmed to not produce detectable DA, regardless of the associated bacterial community. This could indeed imply that the ability to produce DA is more likely an inherent property of the particular *Pseudo-nitzschia* species or strain, rather than an attribute provided by any particular bacterium.

Because DA production was modified in *P. multiseries* cultures, it was possible to link cell physiology to DA production. Cells of *P. multiseries* that did not produce DA were also the cells that grew faster and reached a higher maximal cell concentration. Cultures of *P. multiseries* that produced low amounts of DA exhibited the physiology of healthy cells, even more so than control MM cells, and exhibited inhibition of photosynthesis. Energy acquired was allocated less to primary metabolism (esterase activity) and secondary metabolism (DA production), and less was stored in the form of lipid; thus energy was almost fully allocated to cell division (growth). On the other hand, *P. multiseries* cells with an alien bacterial community produced more DA and did not grow. These cells seemed unhealthy, with photosynthesis and chlorophyll content greatly decreased and a high percentage of dead cells. Cells thus acquired less energy that could be allocated to cell division or stored as lipid. Primary metabolism was enhanced during the first days of growth, as already demonstrated by Lelong *et al.* [19], who showed that the beginning of DA production by *P. multiseries* is associated with an increase in esterase activity. It may thus be assumed that most of the energy, perhaps coming mainly from lipid reserves because photosynthesis was low, was allocated to DA production in *P. multiseries* cultures containing an alien bacterial community. The difference in physiology between MM and MD cultures cannot be explained by bacterial concentration (which was identical), but rather by the species composition of the bacterial community and the number of free-living bacteria per algal cell, which increased greatly in MD cultures as DA production increased. This supports the hypothesis that DA production is linked to some physiological stress induced by the growth of some bacterial communities (specificity yet unknown), while low or no production of DA is linked to healthy cells.

The same experiment was performed with *P. delicatissima* to test whether or not a change in bacterial communities can induce DA production and to confirm any relationship between cell physiological status and DA production. When bacteria were removed, the *P. delicatissima* cultures reached a higher mean cell concentration. In contrast, with an alien bacterial community, *P. delicatissima* cultures reached a lower maximal population density, and no stationary phase was observed, as cells began dying immediately after the exponential phase. Growth rates remained identical regardless of the associated bacterial community. During the exponential phase, there were no differences in the percentages of dead and active cells between culture conditions. *P. delicatissima*, however, seemed healthier without added bacteria. Furthermore, physiological parameters were not different between DM and DD cultures, implying that the composition of the bacterial community did not affect *P. delicatissima* cell physiology measurably. Different species of bacteria have induced different effects on algal cells, from enhancement to inhibition of algal growth [10]. Grossart and Simon [10] found that the addition of a natural bacterial community to axenic *Thalassiosira rotula* increased the growth rate, but cells died faster than in control cultures. Our study showed that *P. delicatissima* cells did not produce DA and cell physiology was not modified upon addition of alien bacteria. The *P. delicatissima* culture used in this latter experiment was likely less stressed, and/or developed some mechanisms/strategies, other than DA production, to compete with the alien bacteria or to counteract negative effects of the bacteria during the exponential phase.

It thus appears that under the tested conditions, production of DA did not favor the growth of *P. multiseries* while, on the other hand, the non-toxic *P. delicatissima* cells seemed more “resilient” to bacterial exchange. One possible explanation is that *P. multiseries* cells exposed to “alien” bacteria produced DA, diverting energy from cell division, thus lowering population growth rate. The questions remain as to: (i) how some bacterial communities act on *Pseudo-nitzschia* cells to induce DA production and/or (ii) by which mechanisms do *Pseudo-nitzschia* cells respond to these bacterial communities. For instance, the bacterial community associated with *P. delicatissima* could produce more gluconolactone/gluconic acid and induce a higher production of DA by *P. multiseries* [22]. Cells of *P. delicatissima* are non-toxic and thus may not be affected by bacterial gluconolactone/gluconic acid production. Regardless of the reason why cells produced DA, it appears that *P. multiseries* cultures that produced increased amounts of DA were associated with lower division rates and a greater proportion of dead cells. We also observed a negative effect of bacteria from the *P. delicatissima* culture on *P. multiseries* growth. Bacteria associated with *P. delicatissima* had no effect on parent culture growth, which could result from co-adaptation in the cultures. This was not the case with *P. multiseries*, which had not grown previously in the presence of the bacterial community from *P. delicatissima*.

Results from this study showed modifications of algal cell physiology and growth resulting from a bacterial exchange, as well as changes in DA production by *P. multiseries*. The *P. delicatissima* culture, however, remained non-toxic after receiving bacteria from the toxic *P. multiseries* culture, which showed that the bacteria, *per se*, did not induce toxicity in the non-toxic species. Further work is needed to better assess the algal physiological responses and identify bacterial species associated with high DA production; this follow-up was unfortunately impossible here as the strains used in this experiment subsequently died.

## 4. Materials and Methods

### 4.1. Culture Conditions

Two species of *Pseudo-nitzschia* were used: *P. multiseries* (Hasle) Hasle (strain CLNN-16, isolated from the Bay of Fundy, Canada) and *P. delicatissima* (Cleve) Heiden (strain Pd08RB, isolated from Brittany, France). Microalgae were cultured in 500-mL flasks containing 250 mL of sterilized f/2 medium [25], at 15.6 °C with an irradiance of 130 μmol photons m^−2^ s^−1^ and a dark:light cycle of 12:12 h. Cultures initially were xenic and grown without antibiotics. Before each sampling, cultures were homogenized gently.

### 4.2. Removal of Bacteria

Before the experiment, each culture of *P. multiseries* and *P. delicatissima* was divided into two different cultures, one treated with antibiotics (AB) and one untreated. The *P. delicatissima* culture was much more resistant to AB than was *P. multiseries* and therefore was exposed to 2 mg mL^−1^ of penicillin and 1 mg mL^−1^ of streptomycin (2:1 of P:S) for 2 day. *P. multiseries* was exposed to 0.2:0.1 mg mL^−1^ of P:S and 0.02 mg mL^−1^ of ampicillin for 4 day (higher concentrations of AB killed both cells and bacteria). At the end of the treatment with AB, *Pseudo-nitzschia* cultures were washed three times by centrifugation (10 min, 16 °C, 800 g) to remove all AB, and the pellet was resuspended into the same volume of f/2 medium. Even though AB treatment greatly reduced the amount of bacteria, cultures exposed to AB were not fully axenic and remaining bacteria were counted during each experiment (see Section 4.4).

### 4.3. Experiments

Experiments started two days after the end of the AB treatment when cells were in the exponential growth phase. For each species, cells were cultured under three conditions, each in triplicate: (i) the AB-treated culture (low bacteria); (ii) the AB-treated culture + bacteria of *P. multiseries*; and (iii) the AB-treated culture + bacteria of *P. delicatissima* (Table 2). For both algae, the control treatment was considered to be the AB-treated culture to which previously associated bacteria were added. Free-living bacteria were obtained after centrifuging (10 min, 16 °C, 800 g) the untreated cultures and sampling the supernatant. Bacteria in these supernatants were counted using a flow cytometer, and it was verified that no microalgal cells remained. The *P. multiseries* experimental flasks were inoculated with 4000 cells mL^−1^ of AB-treated *P. multiseries* (i) alone (low bacteria, M0); (ii) with ~200,000 bacteria mL^−1^ from the untreated *P. multiseries* culture (MM, *P. multiseries* control); or (iii) with ~200,000 bacteria mL^−1^ from the untreated *P. delicatissima* culture (MD). The *P. delicatissima* experimental flasks were inoculated with 2500 cells mL^−1^ of AB-treated *P. delicatissima* (i) alone (D0); (ii) with ~100,000 bacteria mL^−1^ from the untreated *P. delicatissima* culture (DD, *P. delicatissima* control); or (iii) with ~100,000 bacteria mL^−1^ from the untreated *P. multiseries* culture (DM). The bacterial concentration was chosen to remain consistent with the bacteria/*Pseudo-nitzschia* ratio of the untreated cultures of each species. Samples were taken almost daily for cell counts and physiological measurements.

**Table 2:** Experimental design.

	Algal Species Treated with Antibiotics	*P. multiseries* “M-”	*P. delicatissima* “D-”
Bacteria Added			
**No bacterial addition “-0”**		M0	D0
**Bacteria from *P. multiseries* “-M”**		MM (*P. multiseries* control)	DM
**Bacteria from *P. delicatissima* “-D”**		MD	DD (*P. delicatissima* control)

### 4.4. Physiological Measurements

Cell counts and physiological measurements of *Pseudo-nitzschia* spp., quantification of free-living bacteria associated with each *Pseudo-nitzschia* species, and percentage of dead bacteria in the culture were assessed using flow cytometry according to Lelong *et al.* [17]. The physiological measurements were performed with a FACScalibur (BD Biosciences, San Jose, CA, USA) flow cytometer equipped with an argon blue laser (488 nm). To allow comparison between days, the same settings were used for the duration of the experiment. *Pseudo-nitzschia* cells were detected by their FL3 fluorescence. All samples were analyzed for 45 s and cell concentrations were calculated from the flow rate of the flow cytometer, measured daily [26]. Specific growth rate μ (day^−1^) was determined by linear regression of the natural log (cell concentration) *versus* time.

Algal concentration, morphology (Forward Scatter “FSC” as a proxy of size and Side Scatter “SSC” as a proxy for internal complexity), as well as red fluorescence “FL3” (proxy for chlorophyll fluorescence) of algal cells, were measured without any staining. The percentage of dead algal cells was assessed after staining the cultures for 30 min with 0.1 μM of SYTOX Green (Molecular Probes, Invitrogen, Eugene, OR, USA). The intracellular lipid content of algal cells was assessed by staining lipids with 10 μM of BODIPY 493/503 (Molecular Probes, Invitrogen, Eugene, OR, USA) for 30 min. The activity of the primary metabolism of algal cells was assessed after staining with 3 μM of fluorescein diacetate (FDA, Molecular Probes, Invitrogen, Eugene, OR, USA) for 6 min. A 300-μM working solution of FDA was prepared fresh before each experiment. SYTOX Green stains permeable cells (considered to be dead) and FDA stains only cells with active esterases (considered to be metabolically active, [16,19,27]). AB treatment can compromise cell membranes without killing them; this is why the percentages of active and dead cells were monitored in parallel.

The quantum yield (QY = (F_m_ − F_0_)/F_m_) of *P. multiseries* and *P. delicatissima* was measured using an AquaPen-C AP-C 100 fluorometer (Photo Systems Instruments, Drasov, Czech Republic). QY is a measure of the efficiency of the photosynthesis at the photosystem II (PSII) level. F_0_ and F_m_ are, respectively, the minimum and maximum fluorescence of cells at 455 nm [28]. The measurement of QY was performed after the cells were dark-adapted for 20 min at 16 °C. FL3 fluorescence (red autofluorescence) of cells, measured by flow cytometry, was used as a proxy for chlorophyll content as it is linearly related to chlorophyll content [29].

Free-living bacterial concentration and percentage of dead bacteria were obtained after staining for 15 min staining with a final concentration of 1/10,000 of the commercial solution of SYBR Green I (Molecular Probes, Invitrogen, Eugene, OR, USA) and with 10 μg·mL^−1^ of propidium iodide (PI) (Sigma, St. Louis, MO, USA). SYBR Green I stains the DNA of all cells; whereas, PI stains only DNA of “dead” cells, which have lost membrane integrity.

DA content was quantified using an ASP ELISA kit (Biosense Laboratories, Bergen, Norway), following the manufacturer’s protocol. Each sample was analyzed with technical duplicates. Cultures were sonicated for 2 min to quantify total DA (cellular + dissolved). Cultures also were filtered (0.22-μm pore-size acetate cellulose filters, Minisart, Sartorius, Göttingen, Germany) to measure dissolved DA. Intracellular DA was obtained by subtracting dissolved DA from total DA.

### 4.5. Statistics

Results were analyzed statistically with multifactor ANOVA, with time and bacterial treatment as independent variables, to test the effect of the treatment (bacteria or not) over time. One-way ANOVA was used to determine after which day the treatments started to have a significant effect on the variable. The test of rank used was the least significant difference (LSD) test. For all statistical results, a probability of *p* < 0.05 was considered significant. Statistical analyses were performed using StatGraphics Plus (Manugistics, Inc, Rockville, MD, USA).

## 5. Conclusions

This study showed modifications of algal growth and physiology resulting from a bacterial exchange, as well as changes in DA production by *P. multiseries*. Results also demonstrated that the bacteria, *per se*, did not induce toxicity in the non-toxic species, as the *P. delicatissima* culture, remained non-toxic even after receiving bacteria from the toxic *P. multiseries* culture. Further research is needed to better identify bacterial species and algal physiological responses associated with high DA production.
